# Overexpression of Testes-Specific Protease 50 (TSP50) Predicts Poor Prognosis in Patients with Gastric Cancer

**DOI:** 10.1155/2014/498246

**Published:** 2014-03-30

**Authors:** Fang Liu, Qinghua Cao, Ni Liu, Changzhao Li, Changxuan You, Chuanxin Liu, Ling Xue, Rongcheng Luo

**Affiliations:** ^1^Cancer Center, Southern Medical University, Guangzhou 510315, China; ^2^Traditional Chinese Medicine-Integrated Hospital, Southern Medical University, Guangzhou 510315, China; ^3^Department of Medical Oncology, Nanfang Hospital, Southern Medical University, Guangzhou 510515, China; ^4^Department of Pathology, The First Affiliated Hospital of Sun Yat-sen University, Guangzhou 510080, China; ^5^Department of Dermatology and Skin Diseases Research Center, University of Alabama at Birmingham, Birmingham, AL 35205, USA

## Abstract

*Purpose*. To investigate the expression of TSP50 protein in human gastric cancers and its correlation with clinical/prognostic significance. *Methods*. Immunohistochemistry (IHC) analysis of TSP50 was performed on a tissue microarray (TMA) containing 334 primary gastric cancers. Western blot was carried out to confirm the expression of TSP50 in gastric cancers. *Results*. IHC analysis revealed high expression of TSP50 in 57.2% human gastric cancer samples (191 out of 334). However, it was poorly expressed in all of the 20 adjacent nontumor tissues. This was confirmed by western blot, which showed significantly higher levels of TSP50 expression in gastric cancer tissues than adjacent nontumor tissues. A significant association was found between high levels of TSP50 and clinicopathological characteristics including junior age at surgery (*P* = 0.001), later TNM stage (*P* = 0.000), and present lymph node metastases (*P* = 0.003). The survival of gastric cancer patients with high expression of TSP50 was significantly shorter than that of the patients with low levels of TSP50 (*P* = 0.021). Multivariate Cox regression analysis indicated that TSP50 overexpression was an independent prognostic factor for gastric cancer patients (*P* = 0.017). *Conclusions*. Our data demonstrate that elevated TSP50 protein expression could be a potential predictor of poor prognosis in gastric cancer patients.

## 1. Introduction

Gastric cancer is the second most common cancer in the world, causing nearly one million deaths annually [1]. Although the incidence of gastric cancer has decreased, it still remains among the leading causes of death from cancer in China [2]. Despite major advances in diagnosis and treatment in the past few decades, gastric cancer remains a major clinical challenge. Prognostic factors for survival are useful in the management of gastric cancer. Many molecular markers including HER2 [3], E-cadherin [4], and Caveolin-1 [5] have been evaluated as candidate prognostic factors in gastric cancer. However, the prognosis for gastric cancer patients still stays poor, and many prognostic factors, which can effectively predict the prognosis of gastric cancer patients, have not been investigated.

Testes-specific protease 50 (TSP50is a testis-specific gene that encodes a protein, which is homologous to serine proteases [6]. Normally, TSP50 protein is specifically expressed in the spermatocytes of testes but abnormally activated and expressed in breast cancer [6, 7]. Currently, TSP50 is considered as a member of cancer/testis antigens (CTAs), which include almost 140 members, such as melanoma antigen-encoding gene-1 (MAGE-1) [8], cancer/testis antigen cancer-associated gene (CAGE) [9], and Opa interacting protein 5 (OIP5) [10]. These proteins are expressed in various types of human cancers including gastric cancer and may serve as tumor markers for clinical prognosis or targets for therapeutic approaches. In this regard, MAGE-1 protein is a predictive marker of poor prognosis in differentiated advanced gastric cancer patients [8]. Nakamura et al. [10] revealed that OIP5 might be a novel immunotherapy target for patients with gastric cancer. However, the expression of TSP50 protein in gastric cancer and its diagnostic and/or prognostic significance has not been elucidated.

In this study, the expression of TSP50 protein was examined in a large number of human gastric cancer specimens and its clinicopathological and prognostic significance was also assessed.

## 2. Materials and Methods

### 2.1. Patients and Tissue Specimens

Formalin-fixed, paraffin-embedded tissues from 334 patients with gastric cancer and corresponding 20 adjacent nontumor cases, who underwent initial surgical resection between January 2001 and October 2006, were randomly selected from the archives of the Department of Pathology, the First Affiliated Hospital, Sun Yat-sen University, Guangzhou, China. The patients were selected based on availability of resection tissue and follow-up data. None of the patients received preoperative radiation or chemotherapy. Postsurgical chemotherapies were performed depending on the severity of the disease and according to the National Comprehensive Cancer Network (NCCN) guidelines. All the samples were collected with patient's informed consent after approval from the Institute Research Medical Ethics Committee of the First Affiliated Hospital, Sun Yat-sen University.

### 2.2. Tissue Microarray Construction

By reviewing H&E stained slides, the tumor containing areas in the corresponding paraffin-embedded samples were localized and used for the construction of tissue microarray (TMA) as described earlier [11]. Briefly, a hollow needle was utilized to punch and remove bipartite cylinders tissue core (1.0 mm in diameter) from selected donor tissue regions. Further, the punched tissue cores were inserted into a recipient paraffin block with a precisely spaced array pattern, using an automatic tissue-arraying instrument (Beecher Instruments, Silver Spring, Maryland, USA). For each sample, two cores from the selected tumor area and one core from adjacent nontumor mucosa were used to construct the TMA.

### 2.3. Immunohistochemical Analysis

Immunohistochemistry (IHC) staining was performed using a standard streptavidin-biotin-peroxidase complex method as described previously [12]. TMA slides were incubated at 4°C in a moist chamber overnight with rabbit polyclonal antibody against human TSP50 (1 : 100, Proteintech, 12574-1-AP). Staining with PBS instead of primary antibody against TSP50 was used as negative control.

The protein expression level of TSP50 was evaluated by microscopic examination of stained tissue slides. TSP50 expression level was determined by integrating the percentage of positive tumor cells and the intensity of positive staining. The intensity of staining was scored as follows: negative (score 0), bordering (score 1), weak (score 2), moderate (score 3), and strong (score 4). We scored the staining extent according to the percentage of positive stained tumor cells in the field: negative (score 0), 0–25% (score 1), 26–50% (score 2), 51–75% (score 3), and 76–100% (score 4). The product of the intensity and extent score was considered as the overall IHC score (values: from 0 to 16). The staining was observed and assessed by two independent pathologists (Qinghua Cao and Ling Xue) without knowing the identity of the samples. If there was a discrepancy in individual evaluations, then the two pathologists reevaluated the slides together to reach a consensus.

### 2.4. Western Blot Analysis

Liquid nitrogen-conserved gastric cancer tissues (3 pairs of gastric cancer and matched adjacent nontumor specimens from 3 patients) were homogenized and lysed in the RIPA buffer on ice. Protein concentrations were determined by the Bradford method using bovine serum albumin as the standard. Equal amounts of tissue lysates (50 *μ*g) were mixed with 4X loading buffer, boiled for 5 minutes, and subjected to SDS-PAGE. Proteins were electrophoretically transferred to PVDF membranes and the nonspecific sites were blocked with 5% (W/V) nonfat-dry milk in TBST (25 mM Tris-HCl, pH 7.5; 150 mM NaCl; 0.05% Tween-20) for 1 h at RT followed by probing with mouse anti-*β*-actin antibody (1 : 1000, Santa Cruz, SC-81178) or rabbit polyclonal antibody against human TSP50. The membranes were incubated for 1 h with HRP-conjugated secondary antibody. The blots were developed with ECL according to the manufacturer's instructions.

### 2.5. Statistical Analysis

Statistical analysis was performed with the SPSS statistical software package (SPSS Standard version 19.0, SPSS Inc.). The association between TSP50 protein expression and clinicopathological features was analyzed by chi square test. For univariate survival analysis, Kaplan-Meier analysis is used. Log-rank test was used to compare different survival curves. The multivariate Cox regression model was used to assess the potential independent prognostic factors and 95% confidence intervals (CI) of hazard ratio (HR). *P* value of <0.05 was considered statistically significant.

## 3. Results

### 3.1. Clinical Characteristics of Gastric Patients

Table 1 lists the characteristics of recruited patients (*n* = 334). The gender ratio of male to female was 2.3 : 1. The median age was 57 years (range: from 25 to 88 years). The average tumor size (maximum diameter) was 5.2 cm (range: from 1 to 18 cm). Histological features were classified into two types: (a) diffuse or undifferentiated type, comprising poorly differentiated, signet-ring cell and/or mucinous adenocarcinomas, and (b) intestinal or differentiated type, consisting of papillary and/or tubular adenocarcinomas [13]. The number of cases in diffuse type was 69 (20.7%), while the number of cases in intestinal type was 265 (79.3%). TNM staging was distributed as follows: I + II, 117 cases, 35.0%; III + IV, 217 cases, 65.0%. Lymph node metastases were diagnosed in 234 cases (70.1%). The follow-up information of 334 patients was collected within the range from 1 to 62 months after surgery.

### 3.2. Expression of TSP50 Protein in Gastric Cancer Tissues and Its Association with Clinicopathological Parameters

**Figure 1:**
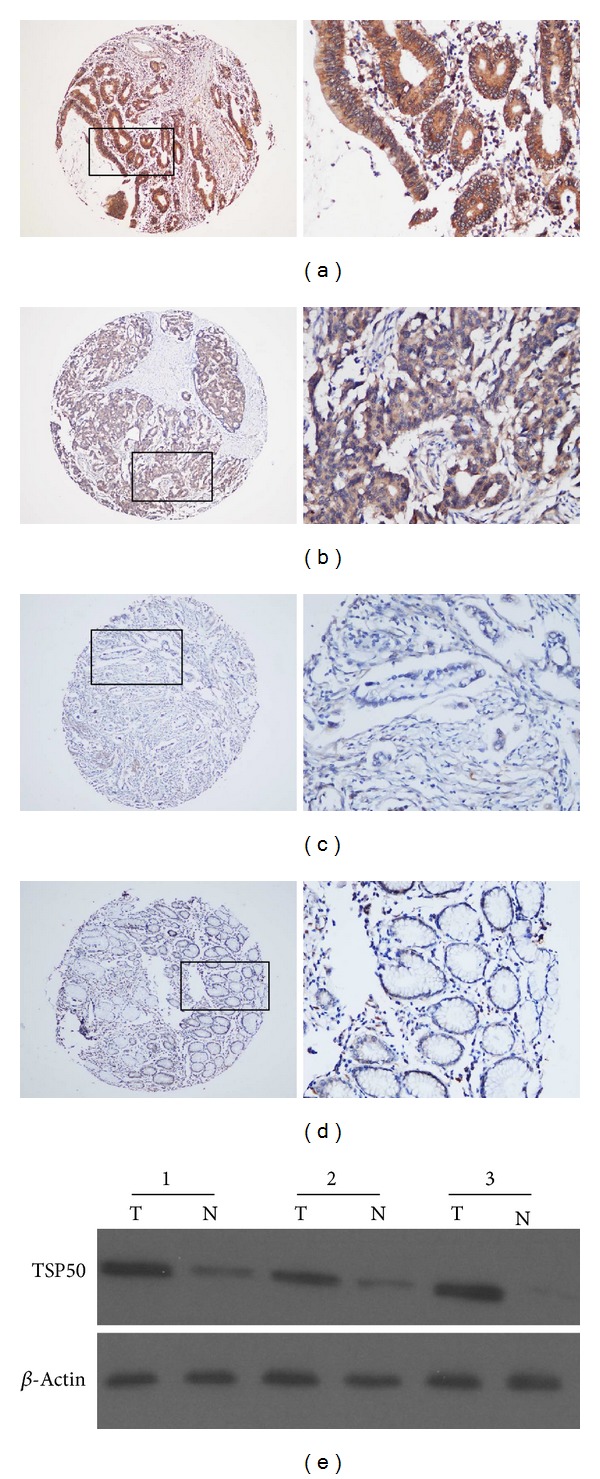
Expression of TSP50 protein in gastric cancer tissues and adjacent nontumor mucosal tissues. Immunohistochemistry (IHC) staining revealed high expression (a, b) and low expression (c) of TSP50 protein in gastric cancers. (a) Scoring index = 16; (b) scoring index = 8; (c) scoring index = 3 (original magnification ×40). The right panel indicated the higher magnification (×400) from the area in (a), (b), and (c), respectively. (d) IHC staining showed low expression of TSP50 protein in adjacent nontumor mucosal tissues (scoring index = 2) (original magnification ×40). The right panel indicated the higher magnification (×400) from the area in (d). (e) Western blot analysis of TSP50 protein expression in gastric cancer tissues (T) and adjacent nontumor mucosal tissues (N). Equal loading of protein was determined by *β*-actin.

Brown membranous and cytoplasm immunoreactivity for the TSP50 protein were recognized as positive staining. The protein expression with a scoring index of ≥8 (median score of TSP50 expression in the gastric cancers) was defined as high expression according to the staining index as mentioned above. High expression of TSP50 was detected in 191 out of 334 gastric cancers (57.2%) (Figures 1(a) and 1(b)) while the remaining 143 cases and all of 20 adjacent nontumor tissues showed only low expression of TSP50 (Figures 1(c) and 1(d)). Western blot analysis also showed that all of 3 gastric cancer samples had significantly higher levels of TSP50 protein than adjacent nontumor tissues (Figure 1(e)). The association between TSP50 expression in gastric cancers and several clinicopathological variables was assessed and displayed in Table 1. The high expression of TSP50 in gastric cancers showed a highly significant relationship with junior age at surgery (*P* = 0.001), later TNM stage (*P* = 0.000), and present lymph node metastases (*P* = 0.003).

### 3.3. Gastric Cancer Patient Survival and Its Relationship with Clinicopathological Features and TSP50 Protein Expression

**Figure 2:**
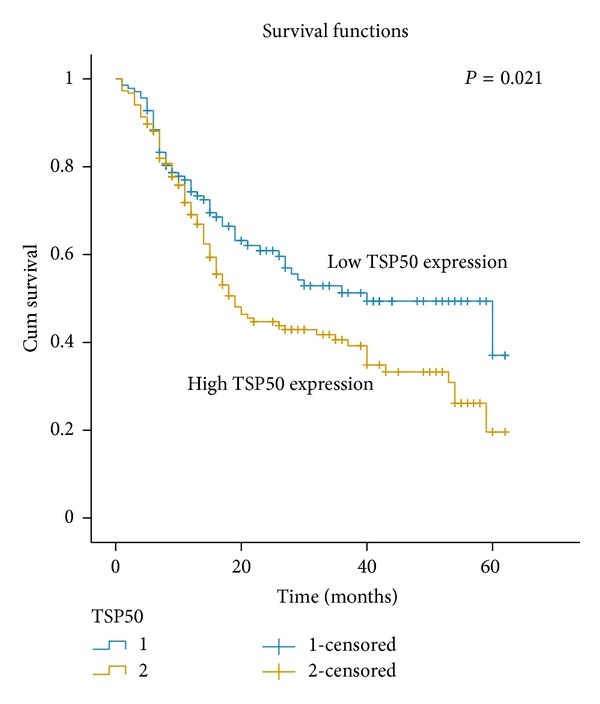
Survival curve for 334 gastric cancer patients according to TSP50 protein expression status (log-rank test). High expression of TSP50 protein was closely correlated with inferior overall survival (OS) (*P* = 0.021).

In univariate survival analysis, Kaplan-Meier survival curves were employed and the statistics were carried out by log-rank method. Kaplan-Meier analysis demonstrated a significant impact of well-known clinicopathological prognostic features such as histological type (*P* = 0.006), TNM (*P* = 0.000), and lymph node metastases (*P* = 0.003) on the survival of gastric cancer patients (Table 2). Furthermore, overall survival was significantly impaired in patients with high expression of TSP50 compared to patients with low expression of TSP50 in tumors (*P* = 0.021). In this regard, the mean value of overall survival time was 38.02 months in patients with low expression of TSP50 compared to 30.21 months in patients with high levels of TSP50 (Table 2, Figure 2).

### 3.4. Prognostic Significance of TSP50 Expression in Gastric Cancer

Multivariate analysis was performed using Cox regression model. High expression of TSP50 as well as other clinicopathological variables (histological type, TNM stage, and lymph node metastases), which were significantly correlated with TSP50 expression, was included in the multivariate analysis. High expression of TSP50 protein was identified as an independent prognostic factor for poor overall survival in patients with gastric cancer (*P* = 0.017, Table 3).

## 4. Discussion

The gene of TSP50 was discovered in a hypomethylated DNA fragment isolated from human breast cancer cells [6], which encodes a testis-specific protease negatively regulated by p53 [14]. It has been shown that TSP50 plays an important role in proliferation and tumor development [15]. Recently, overexpression of TSP50 was shown to be associated with poor prognosis in colorectal cancer [16]. Increasing evidence supports that TSP50 is an oncogene and may serve as a novel biomarker in several types of human epithelia tumors. However, the expression pattern of TSP50 in gastric cancer has not been well established, and its clinicopathological and/or prognostic significance in gastric cancer remains unknown.

In the present study, IHC staining for TSP50 was performed in a large cohort of gastric cancer patients. Our results clearly showed that TSP50 was overexpressed in human gastric cancers. Further analysis showed that high expression of TSP50 is a novel independent factor for poor prognosis in gastric cancer. These results are in line with previous findings in which high levels of TSP50 were shown to be an indicator of poor prognosis in colorectal cancer. However, there was no significant association between TSP50 overexpression and any of the clinicopathological features in that study [16]. In our study, TSP50 overexpression is significantly related to some of the important clinicopathological parameters including junior age at surgery, later TNM, and present lymph node metastases suggesting that TSP50 may play an important role in the development of gastric cancer and tumor metastasis as well.

CTAs, also known as cancer germline antigens, refer to a growing body of tumor antigens [17], which were normally expressed in testis but were aberrantly expressed in tumors of different histological origins. Currently, there are about 70 families of CTAs comprised of 140 members including TSP50 [6, 18, 19]. Although the exact functions of many of these antigens remain unknown, several studies showed that they may contribute to cell cycle progression/regulation, transcriptional control, cell survival, and apoptosis [20–23]. Consistently, knockdown of TSP50 in mouse P19 cells can inhibit tumor cell proliferation and induce apoptosis [24]. The exact mechanism by which TSP50 is involved in tumor development and metastasis remains to be investigated. Recent published data indicate that the interaction between TSP50 and the NF-*κ*B/I*κ*B*α* complex is necessary for TSP50 to perform its function in cell proliferation [25]. These findings highlight the indispensable role of NF-*κ*B signaling in TSP50 mediated tumorigenesis. Since NF-*κ*B is involved in a broad range of pathobiological events including tumor metastasis, one cannot exclude the possibility that TSP50 may contribute to tumor metastasis through NF-*κ*B signaling as well.

Some antigens of CTAs family have also been proved to be novel biomarkers for various types of malignancies, such as MAGE-1 for differentiated advanced gastric cancer [8], LY6K for bladder cancer [26], and sperm protein 17 for cervical cancer [27]. Similar to TSP50, MAGE-1 also serves as an independent prognostic factor of differentiated advanced gastric cancer. Expression of MAGE-1 is correlated with advanced age, macroscopic infiltrated type, and presence of lymph node metastasis in differentiated advanced gastric cancer. These data suggest that antigens including but not limited to TSP50 and MAGE-1 may have similar functions in gastric cancers in terms of tumorigenesis and/or prognosis [8]. In addition, the activation of MAGE-1 in tumor cells is driven by the demethylation of its promoter [28], a common mechanism for the activation of genes in tumor development [29]. Although the mechanism underlying the upregulation of TSP50 in gastric cancer has not been identified, one can speculate that hypomethylation may be also involved in this process based on previous published reports [30–32].

Many of the CTAs are specifically expressed in tumor cells and cancer stem cells and they are immunogenic [33, 34]. Given the advantages of blood-testis barrier and the lack of human leukocyte antigen (HLA) class I expression on the surface of germ cells, immune therapies against CTAs do not cause autoimmune reaction. Therefore, discovery of novel cancer expressing CTAs has led directly to the development of antigen-specific cancer vaccines, providing a new opportunity for immune therapy of these tumors [34, 35]. In this regard, our results uncovered TSP50's potential to be a therapeutic target for gastric cancer as several other CTAs have been carried out for immunotherapy [35–37].

## 5. Conclusions

In summary, we showed that TSP50 is overexpressed in gastric cancers. Elevated TSP50 protein expression is an independent factor for poor prognosis in gastric cancer patients. TSP50 may serve as a novel potential target for the development of specific vaccines against gastric cancers.

## Figures and Tables

**Table 1:** Correlation of TSP50 protein expression with clinicopathological parameters.

Patients features	All cases	TSP50 protein expression		
		High expression	Low expression	*P* value^a^
Gender				
Male	233	138 (59.2%)	95 (40.8%)	0.252
Female	101	53 (52.5%)	48 (47.5%)	
Age at surgery				
≥57^b^	172	84 (48.8%)	88 (51.2%)	**0.001**
<57	162	107 (66.0%)	55 (34.0%)	
Tumor size				
≥5 cm	149	84 (56.4%)	65 (43.6%)	0.788
<5 cm	185	107 (57.8%)	78 (42.2%)	
Histological type				
Intestinal	265	142 (53.6%)	99 (46.4%)	0.582
Diffuse	69	38 (55.1%)	31 (44.9%)	
TNM				
I + II	117	49 (41.9%)	68 (58.1%)	**0.000**
III + IV	217	142 (65.4%)	75 (34.6%)	
Lymph node metastases				
Present	234	146 (62.4%)	88 (37.6%)	**0.003**
Absent	100	45 (45.0%)	55 (55.0%)	

^a^Chi square test; ^b^median age.

**Table 2:** Clinicopathological features and expression of TSP50 protein for prognosis of 334 patients with gastric cancers by univariate survival analysis (log-rank test).

Clinicopathological features	All cases	Mean ± SE^a^	Median ± SE	*P* value
Gender				
Male	233	35.66 ± 1.87	40.00 ± 8.07	0.053
Female	101	28.83 ± 2.59	19.00 ± 2.12	
Age at surgery				
≥57	172	32.34 ± 2.14	27.00 ± 5.63	0.312
<57	162	35.16 ± 2.20	30.00 ± 11.72	
Tumor size				
≥5 cm	149	31.42 ± 2.21	20.00 ± 3.23	0.289
<5 cm	185	35.35 ± 2.11	40.00 ± 6.83	
Histological type				
Intestinal	265	35.04 ± 1.68	30.00 ± 5.15	**0.006**
Diffuse	69	24.81 ± 3.22	13.00 ± 2.22	
TNM				
I + II	117	52.14 ± 2.15	NR^b^	**0.000**
III + IV	217	24.73 ± 1.64	16.00 ± 1.29	
Lymph node metastases				
Present	234	25.73 ± 1.61	17.00 ± 1.31	**0.003**
Absent	100	54.34 ± 1.53	27.00 ± 4.70	
TSP50 protein expression				
Low	143	38.02 ± 2.36	40.00 ± 9.11	**0.021**
High	191	30.21 ± 1.97	19.00 ± 2.32	

^a^SE: standard error; ^b^NR: not reach.

**Table 3:** Multivariate analysis on overall survival (Cox regression model).

Variable	Hazard ratio	95% confidence interval	*P* value
TSP50^a^	1.530	1.078–2.172	0.017
Histological type^b^	1.507	1.004–2.262	0.048
TNM^c^	1.862	1.348–2.573	0.000
Lymph node metastases ^d^	0.286	0.139–0.590	0.001

^a^High expression versus low expression; ^b^intestinal type versus diffuse type; ^c^stages I + II versus stages III + IV; ^d^absent versus present.
